# Primary care surveillance for acute bloody diarrhea, Wales.

**DOI:** 10.3201/eid0604.000418

**Published:** 2000

**Authors:** R M Chalmers, R L Salmon

**Affiliations:** Public Health Laboratory Service Cryptosporidia Reference Laboratory (Wales), Swansea, United Kingdom. roland.salmon@phls.wales.nhs.uk

## Abstract

A sentinel group of primary-care physicians in Wales actively reported cases of acute bloody diarrhea from February 1997 through December 1998. The estimated annual rate was 18 cases per 100,000 population. Most (80%) cases were due to Campylobacter or Salmonella; however, 18% were undiagnosed.


### Primary Care Surveillance for Acute Bloody Diarrhea, Wales

**Rachel M. Chalmers* and Roland L. Salmon†**
*Public Health Laboratory Service Cryptosporidia Reference Laboratory (Wales), Swansea, United Kingdom; †Public Health Laboratory Service Communicable Disease Surveillance Centre (Wales), Cardiff, United Kingdom

A sentinel group of primary-care physicians in Wales actively reported cases of acute bloody diarrhea from February 1997 through December 1998. The estimated annual rate was 18 cases per 100,000 population. Most (80%) cases were due to *Campylobacter* or *Salmonella;* however, 18% were undiagnosed.

The number of cases of acute bloody diarrhea in the United Kingdom and elsewhere is unknown. In addition, while some established etiologic agents of acute bloody diarrhea (*Salmonella*, *Campylobacter*, *Shigella,* and Shiga toxin-producing *Escherichia coli* [STEC] O157) are routinely detected in local diagnostic laboratories, others, such as non-O157 STEC, are not and therefore would not be ascertained by current surveillance. In the United Kingdom, all residents are registered with a general practitioner (a primary-care physician). Since blood in the stool is disturbing to patients, they would be likely to report it to their physicians. We therefore used surveillance by a sentinel group of volunteer general practitioners to estimate cases of acute bloody diarrhea in Wales, detect any clusters, and identify the etiologic agents.

An established network of 34 volunteer general practitioners' offices in Wales (combined registered practice population 223,465 in 1998, representing 8% of the 2,933,324 population) routinely reports cases of measles, mumps, rubella, shingles, chickenpox, influenza, whooping cough, and infective gastroenteritis to the Public Health Laboratory Service Communicable Disease Surveillance Centre (CDSC) (Wales) (1,2). Acute bloody diarrhea was introduced as a reporting category in February 1997, with a clinical case definition of "diarrhea and visible blood of acute onset (first episode only)." Reporting was weekly by paper; data items recorded were age, sex, practice name, and report date. From February 1997 to December 31, 1998, stool samples, requested from the patient by the general practitioner, were submitted to the local clinical diagnostic laboratory. Bacteriologic tests were performed for *Salmonella, Campylobacter, Shigella*, and STEC O157 by culture; ova and parasites were examined by microscopy. Laboratories were contacted for results.

Address for correspondence: Roland Salmon, PHLS CDSC (Wales), Abton House, Wedal Road, Cardiff CF14 3QX, United Kingdom; fax: 441-222-521-987; e-mail: roland.salmon@phls.wales.nhs.uk.

Data were analyzed by person, time, and place. Clusters were defined as three or more cases of acute bloody diarrhea reported in any 2 consecutive weeks by the same practice. (A practice is required, contractually, to specify to the health authority a defined geographic area where all its registered patients should reside.) We used the Poisson distribution to calculate 95% confidence intervals (CIs) for the number of cases; rates were calculated by using the combined practice population of 223,465 as the denominator.

A total of 81 cases of acute bloody diarrhea were reported during the 23-month study period (mean annual rate, 18 per 100,000); 31 cases (95% CI = 21 to 44) were reported from February through December 1997, and 50 cases (95% CI = 37 to 65) during 1998. The age-sex distribution was similar each year, with the highest incidence in young children ages ≤4 years, particularly girls, and the lowest in adults over 65 years. The age distribution differed by sex: rates did not vary in male patients >5 years of age but were high in 25- to 44-year-old women (Table 1). No seasonal distribution pattern was noted.

Table 1. Age and distribution of cases (and annual rates per 100,000 practice population with 95% confidence intervals) of acute bloody diarrhea reported by sentinel general practitioners in Wales, 1997–98

| Age | Total | Males | Females |
|---|---|---|---|
| ≤4 | 15 (63:35-104) | 6 (50:18-109) | 9 (76:35-144) |
| 5-14 | 9 (16:7-30) | 4 (14:14-36) | 5 (18:6-42) |
| 15-24 | 6 (12:4-26) | 4 (15:4-38) | 2 (8:1-29) |
| 25-34 | 15 (25:14-41) | 5 (16:5-37) | 10 (33:16-61) |
| 35-44 | 14 (24:13-40) | 4 (14:4-36) | 10 (34:16-63) |
| 45-64 | 15 (13:7-21) | 6 (11:4-24) | 9 (16:7-30) |
| 65+ | 7 ( 9:4-19) | 3 (9:2-26) | 4 (8:2-20) |
| Total | 81 (18:14-22) | 32 (15:10-21) | 49 (22:16-29) |

In 1997, three clusters were identified, one with four cases, and two with three cases each. Within the first cluster, *Campylobacter* was isolated in two cases, but in the other two clusters both *Salmonella* and *Campylobacter* were isolated. General practitioners' records did not show links between cases within these clusters. In 1998, two clusters were identified. In the first, five persons had *Salmonella;* three patients belonged to the same family, but the other two were not linked to the family or to each other. The second cluster was composed of two sisters with *Salmonella* and two unlinked cases, one *Salmonella* and one *Campylobacter*.

Stool specimens were obtained from 74 (91%) of 81 patients with acute bloody diarrhea. *Salmonella* organisms were isolated from 30 (41%) patients, 11 male and 19 female, ages 8 months to 74 years. *Campylobacter* was isolated from 29 (39%) patients, 13 male and 16 female, ages 1 month to 65 years. All five cases of acute bloody diarrhea in men ages 25-34 years were *Campylobacter*. *Shigella* was isolated in one case, a woman in the 35- to 44-year age group. STEC O157 was not detected (upper 95% CI = 3.7 cases, equivalent to 1.6 per 100,000 total population in Wales). *Cryptosporidium* was detected in one case. A total of 13 (18%) cases (7 in male, 6 in female patients) were undiagnosed by the local laboratory. Stool specimens in 3 of these cases were sent to the Laboratory of Enteric Pathogens, Central Public Health Laboratory, Colindale, London, for testing for other STEC serogroups; none was detected.

This is the first measurement of cases of acute bloody diarrhea in Wales. Since the study sample is broadly representative of the Welsh population (2), the mean annual rate of 18 per 100,000 practice population would equate to 530 cases in Wales as a whole. While a sentinel practice-based scheme cannot measure the true prevalence, it is an economical and efficient way to estimate the order of magnitude of the disease. The low frequency of acute bloody diarrhea and the rarity with which it is encountered by any individual practitioner limit greater precision. The range calculated from the 95% CIs in the study extrapolates to 275 to 850 cases per year in Wales. The difference between the number of cases reported during 11 months in 1997 and the whole of 1998 is not statistically significant since the 95% CIs overlap, but some increase may have occurred as reporting became more efficient.

A high proportion of patients (91%) supplied stool specimens, compared with 67% in a general practitioner-based survey of patients with nonbloody diarrhea (3). *Campylobacter* and *Salmonella*, known causes of bloody diarrhea, were isolated from 80% of specimens, and the epidemiologic characteristics of cases of acute bloody diarrhea frequently reflected those of these organisms (4). For example, the higher annual incidence of acute bloody diarrhea in children <5 years of age (63 per 100,000, compared with 18 per 100,000 for the entire study population) is reflected in laboratory reports to the CDSC (Wales) during the study period (i.e., for *Campylobacter*, an annual incidence of 130 per 100,000, with an incidence in children <5 years of age of 234 per 100,000; for *Salmonella,* an incidence of 68 per 100,000, with an incidence in children <5 years of age of 156 per 100,000 (CDSC [Wales], unpub. data).

One anomaly, however, is the higher rates of acute bloody diarrhea in 25- to 44-year-old women. This is largely accounted for by higher rates of salmonellae (Table 2), something not

Table 2. Cases of acute bloody diarrhea[a] reported by sentinel general practitioners, Wales, 1997–98, by etiologic agents and age

| | *Salmonella* | | *Campylobacter* | | No organism found | |
|---|---|---|---|---|---|---|
| Age | M[b] | F | M | F | M | F |
| ≤4 | 3 | 4 | 2 | 3 | 1 | 1 |
| 5-14 | 2 | 1 | 1 | 3 | 1 | 0 |
| 15-24 | 1 | 2 | 2 | 0 | 1 | 0 |
| 25-34 | 0 | 4 | 5 | 4 | 0 | 1 |
| 35-44 | 1 | 2 | 1 | 2 | 1 | 3 |
| 45-64 | 2 | 5 | 2 | 3 | 1 | 1 |
| 65+ | 2 | 1 | 0 | 1 | 1 | 0 |
| Total | 11 | 19 | 13 | 16 | 6 | 6 |

[a]One isolate of *Shigella* was reported in a woman in the 35- to 44-year age group.
[b]M = males; F = females.

seen in routine surveillance of *Salmonella* isolates in this period (CDSC [Wales], unpub. data). If this finding is true, women in this age group may be more likely than men to exhibit acute bloody diarrhea when infected with salmonellae. No cases of STEC O157 were detected through this surveillance system; however, this organism is rare in Wales. The mean annual rate between 1990 and 1998 was 1.6 cases per 100,000; in 46.3% of cases, blood was detected in the stool (5).

Thirteen (18%) cases for which stool specimens were submitted were of unknown etiology. Although noninfectious causes such as inflammatory bowel disease may also be involved, more diagnoses could almost certainly be made if specimens were sent to a reference laboratory. This could provide useful information about the emergence of, for example, non-O157 STECs. Although difficult to diagnose, such organisms are emerging causes of acute bloody diarrhea and the serious sequelae of hemolytic uremic syndrome worldwide (6).

### Acknowledgments

The authors thank Wendy Walls, Ruth Coomber, Richard Lewis, Robert Smith, Geraldine Willshaw, general practitioners' offices participating in Welsh sentinel surveillance, and National Health Service and Public Health Laboratory Service laboratories in Wales and the English borders.

This work was funded by DH grant number 254.

Dr. Chalmers directs the Public Health Laboratory Service Cryptosporidia Reference Laboratory in Swansea, Wales, United Kingdom. She has a research background in veterinary microbiology and protozoology and current interests in the epidemiology of occupational and food- and waterborne diseases.

### References

1. Thomas DR, Salmon RL, Westmoreland D, Palmer SR. Surveillance of influenza: interpreting sentinel general practice rates using contemporaneous laboratory data: opportunities and limitations. J Epidemiol Community Health 1998;52(Suppl 1):28S-31.
2. Palmer SR, Smith RMM. GP surveillance of infections in Wales. Commun Dis Rep 1991;1:R25-8.
3. Palmer S, Houston H, Lervy B, Ribeiro D, Thomas P. Problems in the diagnosis of foodborne infection in general practice. Epidemiol Infect 1996;117:479-84.
4. Skirrow MB. A demographic survey of campylobacter, salmonella and shigella infections in England. Epidemiol Infect 1987;99:647-57.
5. Chalmers RM, Parry SM, Salmon RL, Smith RMM, Willshaw GA, Cheasty T. The surveillance of Vero cytotoxin-producing *Escherichia coli* O157 in Wales, 1990 to 1998. Emerg Infect Dis 1999;5:566-9.
6. Nataro JP, Kaper JB. Diarrheogenic *Escherichia coli*. Clin Microbiol Rev 1998;11:142-201.
